# Exploring research trends in cancer immunotherapy via single-cell technologies: a scientometric perspective

**DOI:** 10.3389/fimmu.2025.1640224

**Published:** 2025-08-22

**Authors:** Zhongxun Li, Qi Han, Zimu Huang, Sen Zhang, Huina Guo, Hongliang Liu, Xiaoya Guan, Hairong Li, Chunming Zhang

**Affiliations:** ^1^ Shanxi Key Laboratory of Otorhinolaryngology Head and Neck Cancer, First Hospital of Shanxi Medical University, Taiyuan, China; ^2^ Shanxi Province Clinical Medical Research Center for Precision Medicine of Head and Neck Cancer, First Hospital of Shanxi Medical University, Taiyuan, China; ^3^ Shanxi Medical University, Taiyuan, China; ^4^ Department of Otolaryngology Head and Neck Surgery, First Hospital of Shanxi Medical University, Taiyuan, China; ^5^ Department of Cell Biology and Genetics, The Basic Medical School of Shanxi Medical University, Taiyuan, Shanxi, China; ^6^ Department of Histology and Embryology, Shanxi Medical University, Taiyuan, China

**Keywords:** immunotherapy, tumor immune microenvironment, heterogeneity, single-cell RNA sequencing, bibliometrix

## Abstract

**Introduction:**

Cancer immunotherapy has brought new therapeutic hopes for cancer patients, but it is complex in its mechanism of action, and there are significant individual differences, which restricts its wide use. As single-cell analysis technology develops rapidly, it provides an innovative research approach to investigate immunotherapy mechanisms, to identify potential biomarkers, and to optimize individualized treatment strategies.

**Methods:**

The Core Collection of Web of Science (WOSCC) was used to retrieve and obtain relevant literature related to “application of single cell sequencing in cancer immunotherapy” since the establishment of the WOSCC. A quantitative analysis and visualization of the related literature was conducted using tools such as Bibliometrix, Excel, CiteSpace, VOSviewer, and Scimago Graphica.

**Results:**

The total number of related literatures included was 4856, with an average annual growth rate of 25.14%. According to published articles, China and the United States lead the field. Frontiers in immunology, Nature Communications, Journal for Immunotherapy of Cancer, Scientific Reports, Frontiers in Oncology and Cancers have an important academic influence in this field. Research hotspots focus on tumor immune microenvironment and cellular heterogeneity. Research trends such as spatial transcriptomics, standardized processes, and T cell function are becoming increasingly popular.

**Conclusion:**

In tumor immunotherapy, single-cell sequencing is profoundly changing the research paradigm. It not only improves our understanding of the immune microenvironment and therapeutic heterogeneity, but also assists us in identifying accurate markers and formulating individualized treatment plans.

## Introduction

Immunotherapy has emerged as one of the most effective cancer treatment strategies, owing to its strong specificity, fewer adverse effects, and capacity to induce immune memory—advantages that set it apart from traditional therapeutic approaches. Notably, the advance immune checkpoint inhibitors and chimeric antigen receptor T cell therapy has markedly enhanced the prognosis of patients with diverse forms of advanced malignant tumors (1–3). Nevertheless, the clinical application of immunotherapy remains challenging due to several limitations, including primary resistance in some patients, rapid relapse following an initial therapeutic response (acquired resistance), and the occurrence of potentially severe immune-related adverse events (4). Due to the complexity of cellular composition and the heterogeneity of functional states within the tumor microenvironment (TME), conventional molecular techniques often fall short in accurately capturing these features.

With unprecedented resolution, single-cell omics technologies—especially single-cell RNA sequencing (scRNA-seq)—have recently enabled detailed characterization of the cellular composition, functional states, and intercellular communication networks of immune cell subsets within TME (5). Through single-cell profiling of tumor-infiltrating lymphocytes, dendritic cells, and tumor-associated macrophages, researchers have uncovered the dynamic trajectories of T cell exhaustion, immunosuppressive signatures of myeloid populations, and identified novel immune subpopulations and key molecular markers closely associated with immunotherapy efficacy. For instance, Zheng et al. conducted a single-cell sequencing analysis of tumor-infiltrating lymphocytes from hepatocellular carcinoma patients, revealing marked heterogeneity in functional states and clonal expansion, which was strongly correlated with treatment response (6). In another study, Cheng et al. constructed a comprehensive transcriptomic atlas of myeloid cells across multiple cancer types, highlighting the pivotal immunoregulatory roles of LAMP3^+^ dendritic cells and VEGF^+^ macrophages (7). The integration of single-cell multi-omics technologies—such as single-cell ATAC sequencing (scATAC-seq), Cellular Indexing of Transcriptomes and Epitopes by sequencing (CITE-seq), and spatial transcriptomics—is progressively enabling a three-dimensional, dynamic reconstruction of the cancer immune ecosystem. This advancement offers robust support for the design and refinement of precision immunotherapy strategies. As the volume of literature in this domain continues to grow rapidly, there is an increasing need for systematic approaches to delineate research trajectories, identify thematic hotspots, and predict future directions.

Scientometric analysis, which applies mathematical and statistical methods to the study of scholarly literature, offers a systematic perspective on the evolution of specific research fields and unveils their structural characteristics. It enables the visualization of shifting research frontiers and facilitates the quantitative assessment of contributions made by countries, institutions, and individual scholars (8). Moreover, it aids in identifying emerging themes and potential future directions. In recent years, the convergence of single-cell technologies and immunotherapy in oncology has emerged as a prominent interdisciplinary research focus. Accordingly, this study employs scientometric approaches to explore and visualize the research landscape of single-cell analysis in the context of tumor immunotherapy. The goal is to provide researchers with a macro-level understanding of the field’s development, highlight forward-looking research topics and key technological pathways, and ultimately promote advances in both basic science and clinical application.

## Materials and methods

### Data collection and retrieval strategies

Web of Science Core Collection (WoSCC) is a comprehensive academic database covering over 190 subject areas globally. It is widely recognized as the superior database for bibliometric research in various disciplines, providing excellent literature retrieval and citation analysis services (9, 10). A comprehensive search of WOSCC was conducted to identify literature related to single-cell analysis in antitumor immunotherapy, covering all records since the database’s inception. To minimize potential bias due to ongoing updates of the database, all searches were performed on the same day. The search strategy was as follows: TS = (“immune therapy” OR immunotherapy OR immunity OR immunotherapies) AND TS = (cancer* OR tumor* OR carcinoma* OR neoplasm*) AND TS = (“single-cell analysis” OR “single-cell RNA*” OR “single-cell near/3 sequencing” OR “single-cell transcriptomic” OR “single-cell near/3 profiling” OR “single-cell omics”). The following types of documents were excluded (1): non-English publications; (2) early access articles, book chapters, retracted publications, conference proceedings, and data papers.

A total of 4,856 documents were retrieved for further analysis, including 4,481 research articles and 375 review articles. For each publication, the following metadata were extracted: title, publication year, authors, country/region, affiliated institutions, source journal, keywords (including author keywords and keyword plus), citation count, number of references, references and abstract. Journal impact factors were primarily obtained from the 2023 edition of the Journal Citation Reports (JCR). For journals without JCR data, **Supplementary Information** was retrieved from LetPub (http://www.letpub.com.cn).

### Statistical analysis

The retrieved data were exported in plain text format and subsequently imported into various tools for analysis and visualization, including Biblioshiny (a web interface based on Bibliometrix 4.3.3), VOSviewer (version 1.6.19), CiteSpace (version 6.4.R1), and Microsoft Excel (version 2021 Professional Edition). The processed files contained bibliographic metadata, including titles, publication dates, authors, countries/regions, affiliations, keywords, Keyword Plus, reference counts, cited references, abstracts, and other relevant information. The statistical analysis of this study is based on the comprehensive table (**Supplementary Table S1**).

Bibliometrix (11), an R-based bibliometric analysis and visualization package, was used for statistics and generating strategic maps. These two-dimensional maps position density (indicative of thematic development/maturity) on the vertical axis and centrality (reflecting a theme’s connectivity and relevance to other themes) on the horizontal axis (12, 13). National and author collaboration networks were analyzed using VOSviewer (14). A global distribution map of scientific output by country was created with SCImago Graphica (Beta version 1.0.49) (15). Cytoscape (16) (version 3.10.2) was employed to visualize author collaboration networks. CiteSpace (version 6.4.R1) (17) was employed to conduct a series of advanced bibliometric analyses, including keyword clustering, co-citation reference clustering, extraction of raw data for alluvial diagram construction, co-citation network analysis, and burst detection. Additionally, the software supports integration with the Ollama: llama3 8B model, thereby enhancing the efficiency and intelligence of bibliometric data processing. A modularity Q value greater than 0.3 and a mean silhouette score above 0.5 are generally considered to be indicative of robust and meaningful clustering results.

Visualization outputs, including pie charts, bubble plots, stacked bar charts, radar charts, histograms, line plots, violin plots and heatmaps, were generated using R (version 4.5.0). Statistical significance was determined using a p-value of less than 0.05.

## Results

### Overview of the study status

A total of 4,856 publications related to single-cell analysis in tumor immunotherapy were retrieved from WOSCC, including 4,481 original research articles and 375 review articles. These publications, spanning the years 1998 to 2025, were published across 736 academic journals. The dataset encompasses contributions from 37,912 authors, affiliated with 22,517 institutions in 66 countries. Country attribution was determined based on the institutional affiliations of all contributing authors.

**Figure 1:**
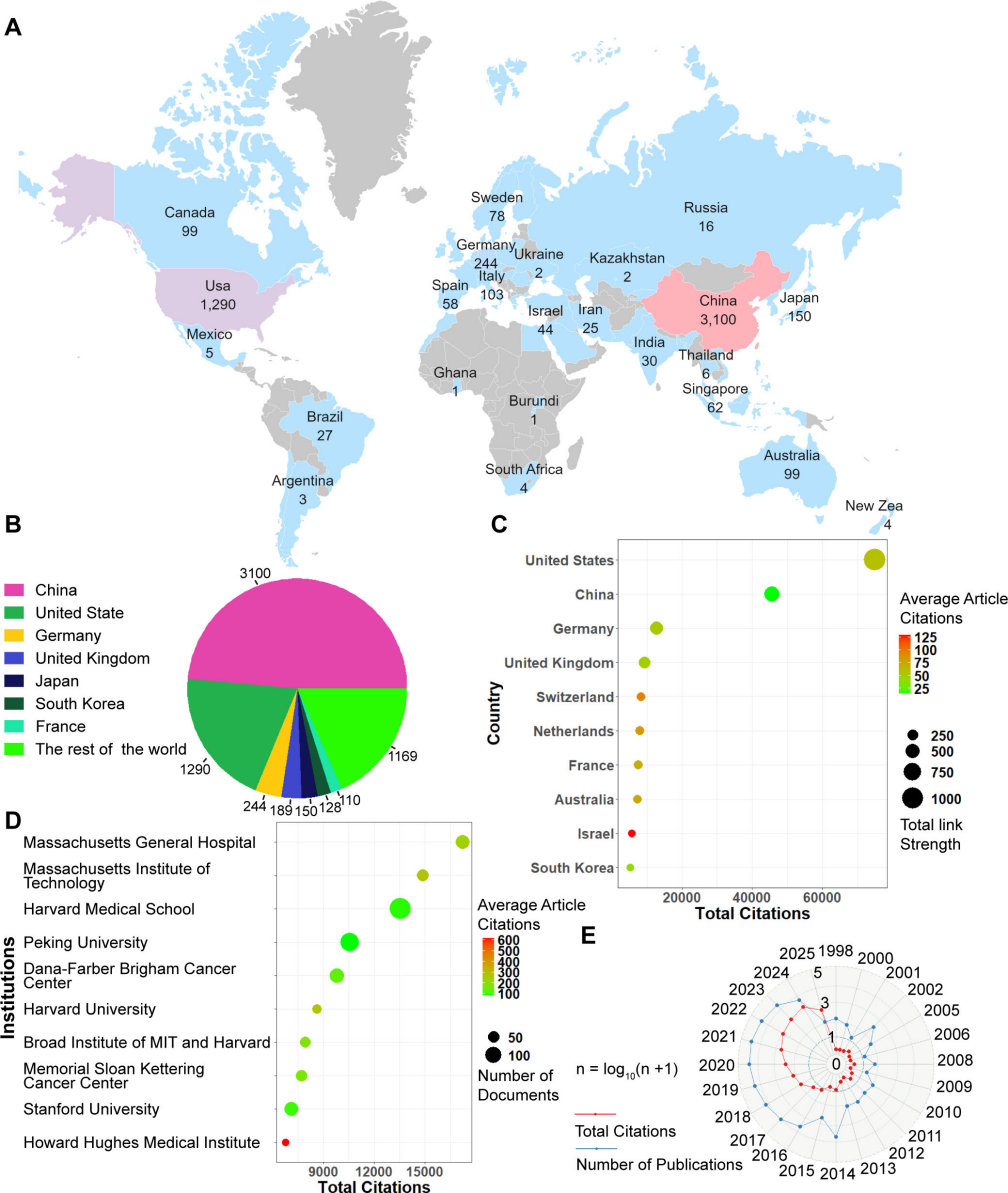
Global publishing overview. **(A)** Global distribution map of publication volume. **(B)** Top 7 countries by number of publications. **(C)** Top7 Countries with total citation. **(D)** Top 7 institutions ranked by total citations. **(E)** Annual trends in publication volume and citation frequency.

**Figure 1A** shows the global distribution of publications. The top seven countries accounted for over 80% of the total number of articles. **Figure 1B** shows that the top seven countries with the highest publication counts were China (3,100), followed by the United States (1,290), Germany (244), the United Kingdom (189), Japan (150), South Africa (128), France (110) and the rest of the world (1169). **Figure 1C** shows the average citations per article and the total link strength for the top 10 most frequently cited countries. The top five countries by total citation count were the United States (74,989 citations), China (45,666), Germany (12,744), the United Kingdom (9,321), and Switzerland (8,261). Country attribution was determined using the full counting method as defined in VOSviewer for all author affiliations.

**Figure 1D** presents the top 10 institutions ranked by total citations, along with their average citations per article and the number of publications. The top 5 most cited institutions were Massachusetts General Hospital, Massachusetts Institute of Technology, Harvard Medical School, Peking University, and Dana-Farber/Brigham and Women’s Cancer Center. Since 2017, the number of publications in this field has increased rapidly (**Figure 1E**). The volume of publications in this field is anticipated to increase steadily in the coming years.

**Figure 2:**
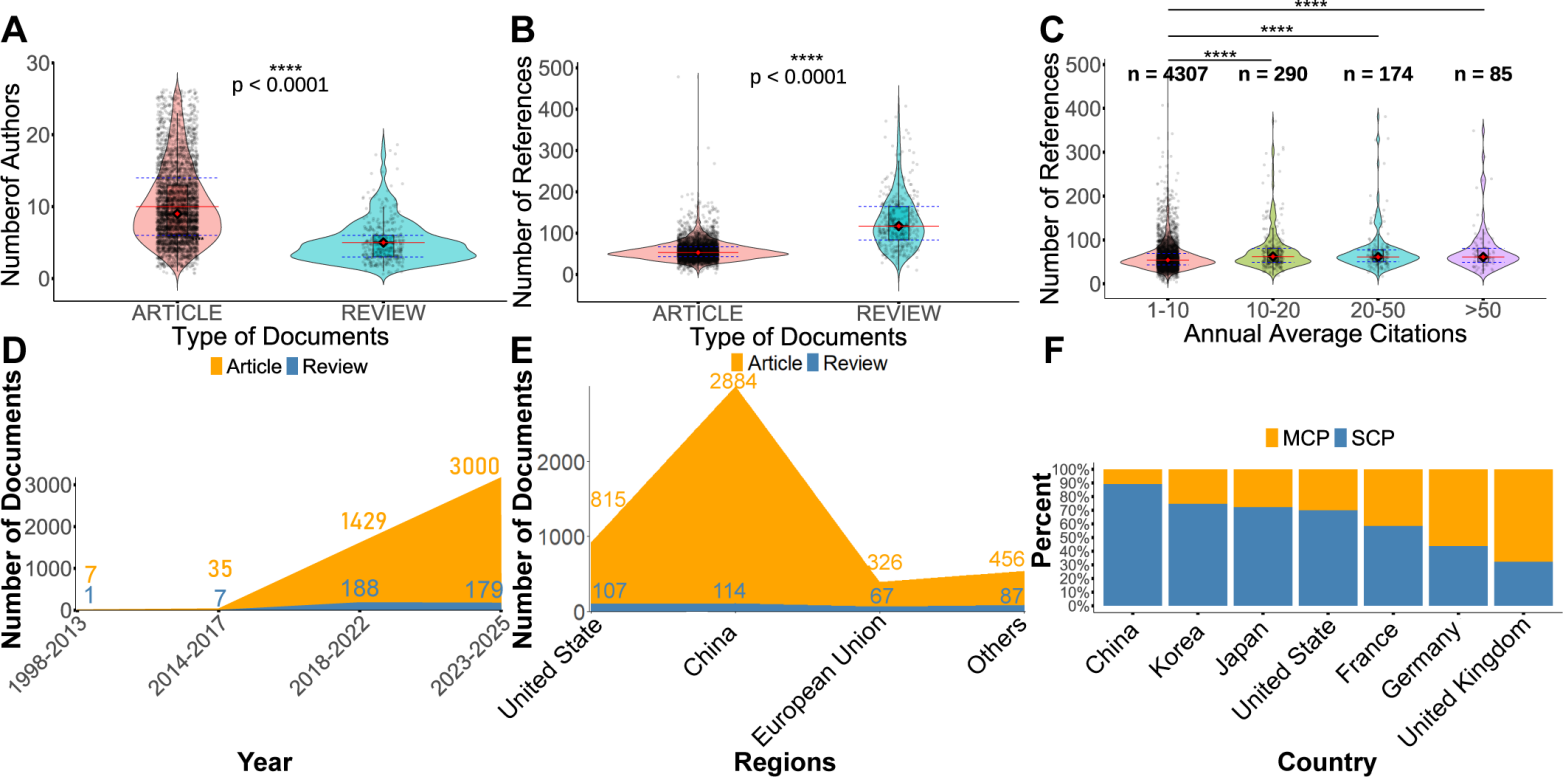
Trends in the publication and citation of articles. **(A)** Comparison of the number of authors per article between research articles and reviews. **(B)** Comparison of the number of references per article between research articles and reviews. **(C)** Distribution of the number of references in articles grouped by different levels of annual average citations. **(D)** Temporal distribution of research articles and review papers across different publication periods. **(E)** Distribution of the number of research articles and review papers published by China, the United States, European Union and other countries. **(F)** Distribution of multiple-country publications (MCP) and single-country publications (SCP) for the top seven countries with the highest volume of SCP. MCP indicates international collaborations, whereas SCP reflects domestic collaborations only. *P < 0.05, **P < 0.01, ***P < 0.001, ****P < 0.0001.

**Figure 2A** shows that the number of authors per research article was significantly higher than that of review articles (*P* < 0.0001). In contrast, **Figure 2B** demonstrates that review articles contained significantly more references than research articles (*P* < 0.0001). A correlation analysis was subsequently performed after categorizing publications by their average annual citation frequency in **Figure 2C**. Articles with an average citation frequency greater than 10 were found to include significantly more references—approximately 10 times the average—than those with lower citation rates.

An analysis of publication trends over time revealed that the number of research articles published before 2017 was relatively low, with a marked increase observed only after that year (**Figure 2D**). Moreover, the number of research articles consistently exceeded that of review articles across all time periods.

To avoid double counting, country attribution was determined by the affiliation of the first author. Given the political, economic, and cultural integration within the European Union, it was treated as a single entity for analytical purposes. Collectively, China and the United States, China and the European Union accounted for 88.8% of all publications,89.8% of research articles and 76.8% of reviews in the field. Furthermore, these three regions published 231.0% more review articles than all other countries combined (**Figure 2E**). Among European countries, the United Kingdom, France, and Germany demonstrated the highest levels of international collaboration, while the United States led in North America (**Figure 2F**). In contrast, China had the highest number of publications produced without international collaboration.

### Author analysis

Given that first authors are often either corresponding authors or key contributors to a publication, we conducted a statistical analysis of first authors in this field using the bibliometrix R package. The h-index of each first author was calculated to assess their academic impact.

**Figure 3:**
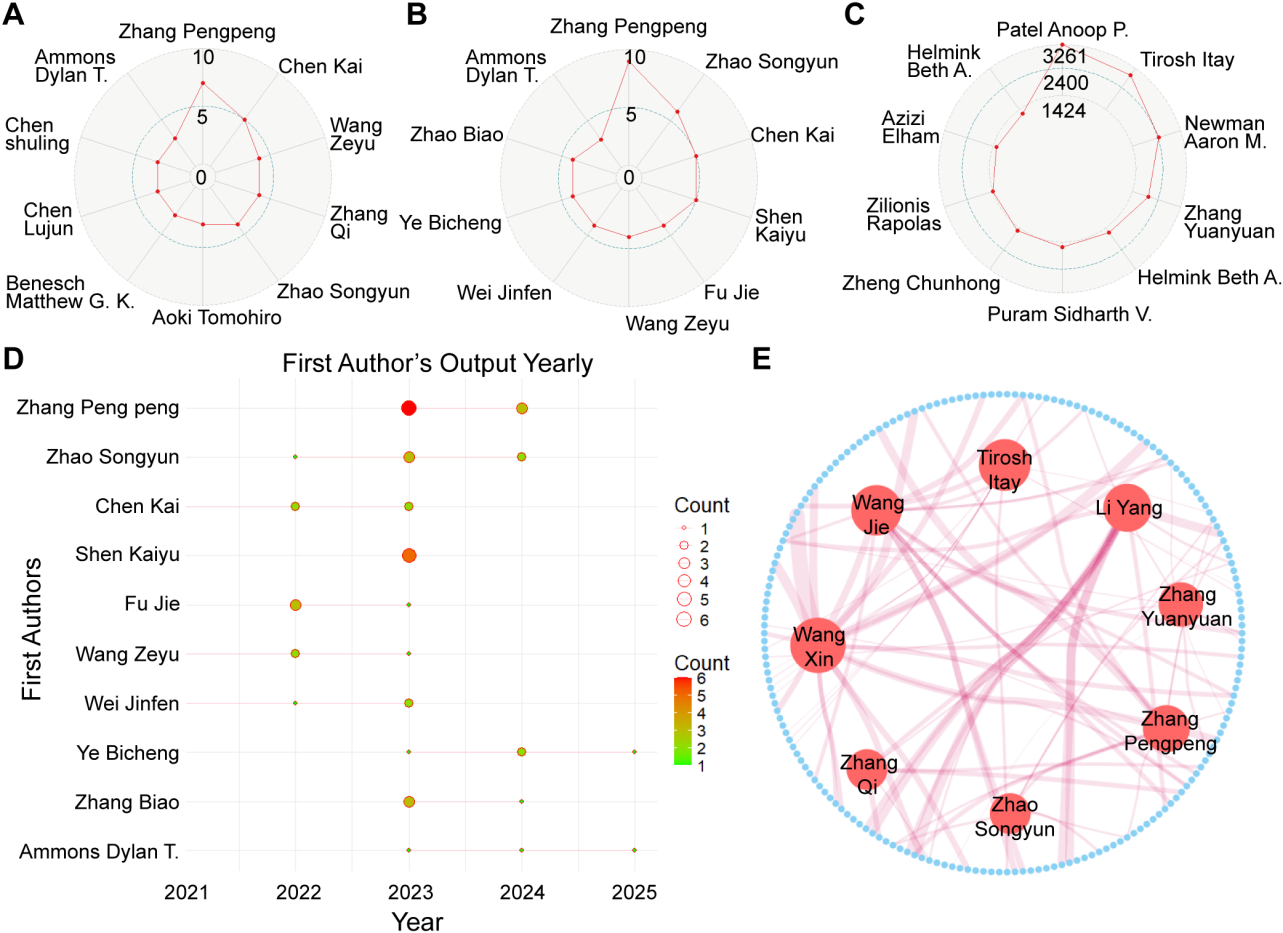
Author analysis. **(A)** Top 10 first authors ranked by h-index. **(B)** Top 10 first authors with the highest number of publications. **(C)** Top 10 first authors with the highest total citation counts. **(D)** Annual publication trends of the top 10 most prolific first authors. **(E)** Co-authorship network of authors based on the top 200 standardized citation frequencies.

**Figure 3A** presents the h-indices of the top 10 first authors. Among them, Zhao Pengpeng (h = 7), Cheng Kai (h = 5), and Wang Zeyu (h = 4) exhibited the highest h-index values. As shown in **Figure 3B**, the authors with the highest number of publications as first authors were Zhang Pengpeng (9), Zhao Songyun (6), and Chen Kai (6). **Figure 3D** illustrates the annual publication output of the top 10 first authors, indicating that their relevant publications only began appearing after 2022. **Figure 3C** displays the top authors based on total citation frequency, with Patel Anoop P. (3,261), Tirosh Itay (2,956), and Newman Aaron M. (2,433) leading the list.

It was observed that older publications generally accumulate more citations over time, whereas more recent works have had limited opportunity to do so. Moreover, citation patterns differ markedly across subfields, with certain research areas attracting significantly more scholarly attention. To reduce the confounding effects of publication year and disciplinary variation on citation frequency, a normalized citation metric was employed. Based on the top 200 authors, an author co-citation network was constructed to illustrate intellectual linkages within the field. In addition, an author collaboration network was visualized to identify key contributors. Notably, Zhang Pengpeng, Li Yang, and Wang Jie emerged as central figures in this network (**Figure 3E**). **Table 1** presents detailed information on all aforementioned authors.

**Table 1:** Details of all authors listed, sorted alphabetically.

Author	Institution	Country
Ammons Dylan T.	Colorado State University	United State
Aoki Tomohiro	British Columbia Cancer	Canada
Azizi Elham	Memorial Sloan Kettering Cancer Center	United State
Benech Matthew G. K.	Roswell Park Comprehensive Cancer Center Buffalo	United State
Chen Kai	Peking University	China
Chen Lujun	Third Affiliated Hospital of Soochow University	China
Chen Shuling	Sun Yat-Sen University	China
Fu Jie	Central South University	China
Helmink Beth A.	The University of Texas Md Anderson Cancer Center	United State
Helmink Beth A.	The University of Texas Md Anderson Cancer Center	United State
Newman Aaron M.	Stanford University	United State
Patel Anoop P.	Massachusetts General Hospital and Harvard Medical School	United State
Puram Sidharth V.	Department Of Pathology and Center for Cancer Research	United State
Shen Kaiyu	Zhejiang Chinese Medical University	China
Tirosh Itay	Broad Institute of Mit and Harvard	United State
Wang Zeyu	Central South University	China
Wei Jinfen	South China University of Technology	China
Ye Bicheng	Yangzhou Polytechnic College	China
Zhang Biao	Dalian Medical University	China
Zhang Pengpeng	Nanjing Medical University	China
Zhang Qi	Zhejiang University	China
Zhang Yuanyuan	Peking-Tsinghua Center for Life Sciences	China
Zhao Songyun	Nanjing Medical University	China
Zheng Chunhong	Peking University	China
Zilionis Rapolas	Harvard Medical School	United State

### Country analysis

**Figure 4:**
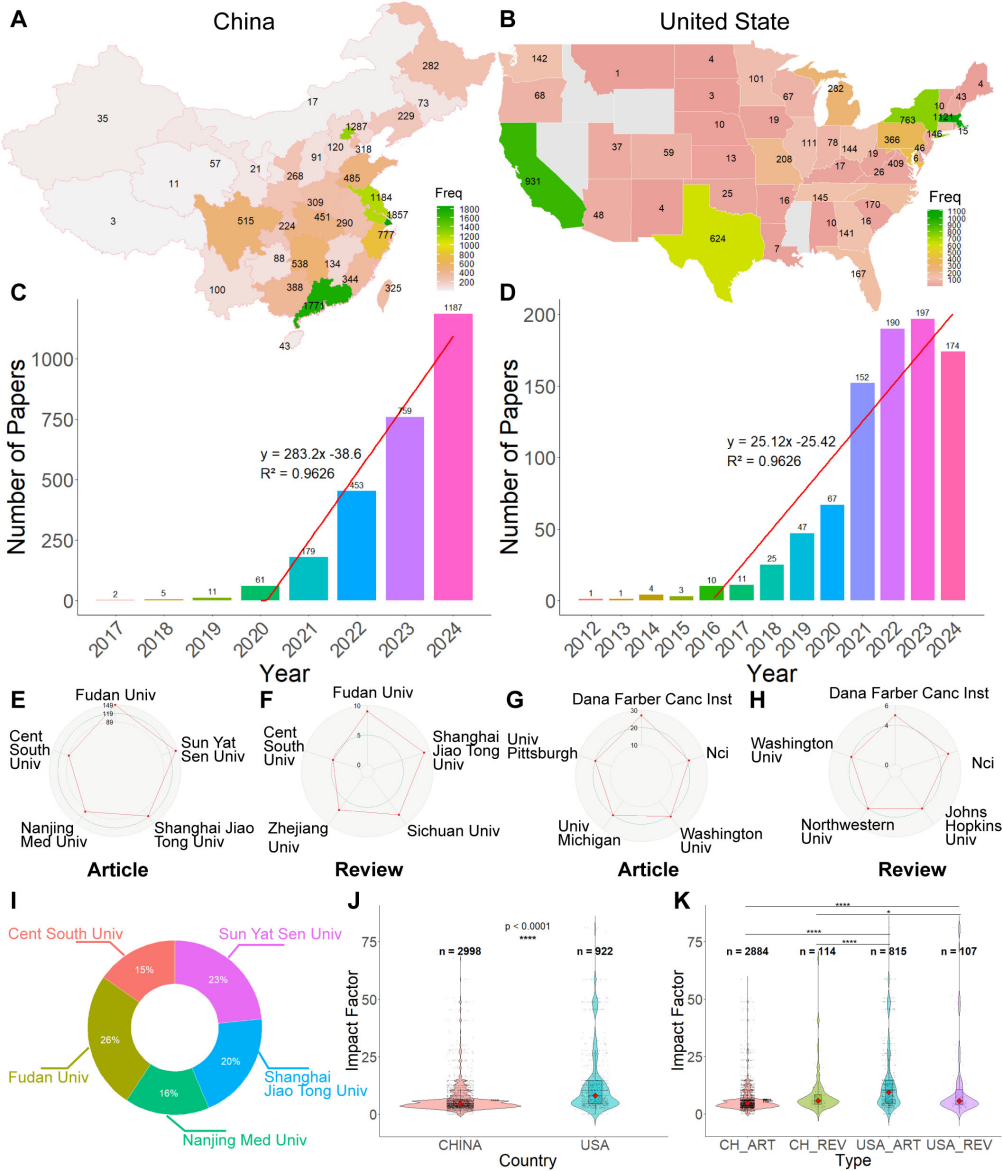
Country-level analysis of publications. **(A)** Frequency distribution of publications by province in China. **(B)** Frequency distribution of publications by United states **(C)** Annual distribution of publications in China. **(D)** Annual distribution of publications in the United States. **(E)** Top 5 institutions in China by number of published research articles. **(F)** Top 5 institutions in China by number of published review articles. **(G)** Poll top 5 organizations with the most published articles in the US **(H)** Top 5 institutions in the United States by number of published review articles. **(I)** Comparison of the top 5 institutions in China and the United States by number of published articles. **(J)** Impact factor distribution of articles published in China and the United States. **(K)** Impact factor comparison of different publication types (research *vs*. review) in China and the United States. *P < 0.05, **P < 0.01, ***P < 0.001, ****P < 0.0001.

We conducted a comprehensive analysis of the countries affiliated with all authors, recording the institutional country information for each article. The results showed that China (12,836) and the United States (6,668) together accounted for 73.46% of all publications frequence (26,550) in this field, highlighting their dominant contribution. Subsequently, we analyzed the regional distribution of publications within China and the United States. **Figure 4A** illustrates the publication output across 32 regions of China, including Taiwan. The distribution is notably concentrated in coastal provinces, which aligns with the higher levels of economic development, better transportation infrastructure, resource availability, and regional clustering effects in these areas. Specifically, Shanghai (1,857), Guangdong (1,771), Jiangsu (1,184), and Zhejiang (777) ranked among the top contributors. In contrast, publication output from inland regions was relatively low. Apart from Beijing (1,287), research productivity across other regions was scattered and uneven, reflecting disparities in regional development and scientific capacity. **Figure 4B** shows the distribution of publications across 47 Unite States. The leading contributors were Massachusetts (1,121), California (931), New York (763), Texas (624), Maryland (409), Pennsylvania (366), Michigan (282), and Missouri (208). These states are characterized by strong economic foundations and a high concentration of scientific, technological, and educational resources, which likely contribute to their high publication output in this field.

Given the variability in the number of authors per article, countries with larger author lists may be overrepresented in publication frequency statistics. To mitigate this bias, we analyzed publications based on the country and institutional affiliation of the first author. Since authors often report multiple affiliations, we considered only the first-listed institution, as it typically reflects the primary workplace and main research output source of the author. In the field of single-cell research and immunotherapy, first authors were affiliated with institutions across 57 countries. Notably, China and the United States together accounted for 3,920 publications, representing 80.72% of the global output (n = 4,856). Given their dominant contribution, we conducted a more detailed analysis of first-author affiliations within these two countries.

Since only one article with a first author from China was published before 2017 (in 2009), we plotted the annual publication trend starting from 2017 and performed a curve fitting analysis (**Figure 4C**). The results show a steady increase in China’s publication output from 2017 onward, with a sharp surge beginning in 2020. The number of publications peaked in 2024, reflecting a remarkable growth rate of 283.2%. This rapid expansion indicates substantial progress in scientific research and collaboration within China, underscoring the country’s increasing impact on global academic output. Continued growth is anticipated in the coming years.

In contrast, the United States began to exhibit an increasing publication trend earlier, with a noticeable rise starting after 2012. Therefore, we analyzed and fitted the annual publication trend of U.S. first authors from 2012 onward (**Figure 4D**). Between 2012 and 2015, publication growth was relatively modest, followed by a significant acceleration beginning in 2016, with an annual growth rate reaching 25.12%. This upward trajectory is expected to persist. Prior to 2012, there were only three relevant publications from first authors in the United States—one each in 2002, 2005, and 2008.

Subsequently a statistical analysis of the publication frequency of institutions affiliated with first authors in China and the United States was conducted. **Figures 4E–H** present the top 5 institutions for original research articles and reviews in each country: Chinese research articles (**Figure 4E**), Chinese reviews (**Figure 4F**), U.S. research articles (**Figure 4G**), and U.S. reviews (**Figure 4H**).

In China, the most prolific institutions were Fudan University (articles: 149; reviews: 9), Sun Yat-sen University (articles: 142; reviews: 3), and Shanghai Jiao Tong University (articles: 116; reviews: 9). In the United States, leading contributors included Dana-Farber Cancer Institute (articles: 27; reviews: 5), the National Cancer Institute (NCI) (articles: 21; reviews: 5), and Washington University (articles: 21; reviews: 4). Collectively, the top 5 institutions from China and the United States accounted for 15.10% of all publications originating from these two countries.

**Figure 4I** summarizes the overall top 5 institutions across both countries. Among them, Fudan University, Sun Yat-sen University, and Shanghai Jiao Tong University ranked as the top 3 contributors, highlighting China’s strong institutional presence in this field.

Afterwards the impact factors of articles published in China and the United States were analyzed. JCR’s latest impact factors for 2023 were used. The impact factors of journals not included in the latest JCR journals SCIE are recorded as 0. The number of articles in China is 32, while the number of articles in the United States is 21. There are 19 magazines that are not included. It was found that the impact factor of USA articles (n = 922) was substantially greater than that of Chinese articles (n = 2998) (p < 0.0001) (**Figure 4J**). In order to further clarify, an analysis chart of the impact factors of Chinese and American articles and reviews was displayed in **Figure 4H** and found that American articles had significantly higher impact factors than those of Chinese articles (p < 0.0001) and reviews (p < 0.0001). The impact factor of the USA review was significantly higher than that of the Chinese article (p < 0.0001) and the review (p < 0.05). This may be related to the large number of articles published in China, including many articles with low impact factors. There were no significant differences in the impact factors between USA articles and reviews. At the same time, there were no significant differences in the impact factors between Chinese articles and reviews.

**Figure 5:**
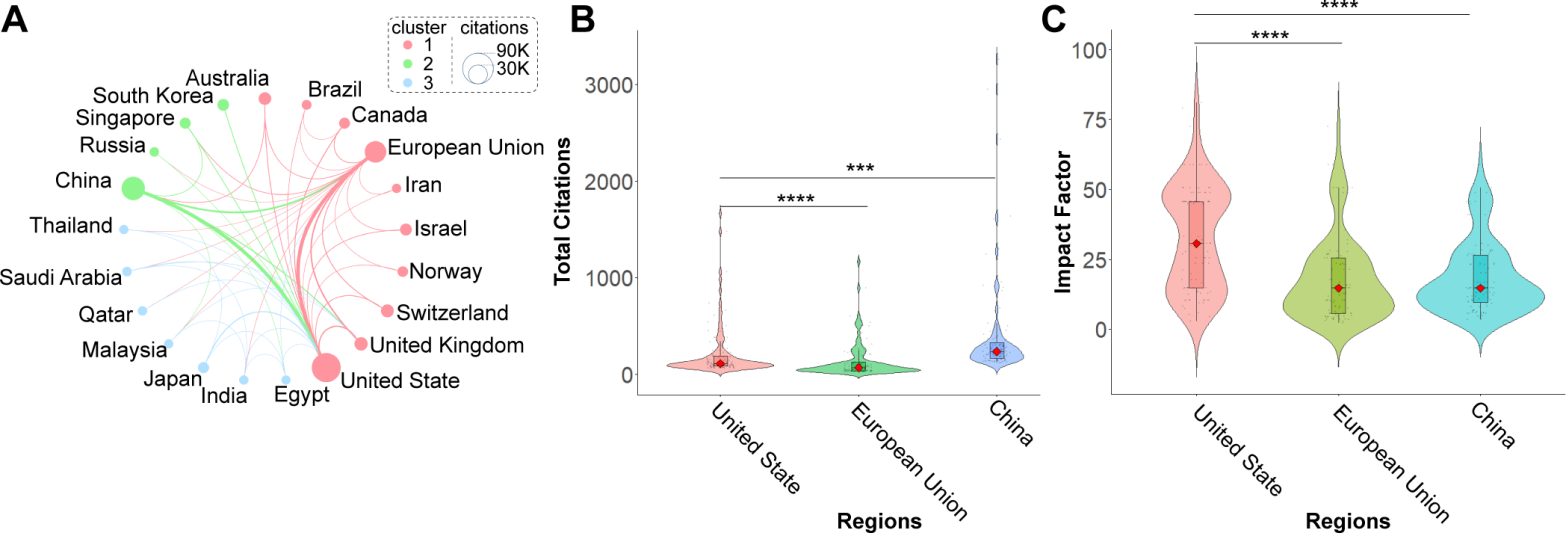
Comparative Analysis of China, the United States, and the European Union. **(A)** Interaction network analysis among the top 21 contributing countries, with the European Union treated as a single entity. Each node represents a country, with node size proportional to its total citation count. Node colors indicate different clusters or collaboration groups. The thickness of the connecting lines reflects the strength of international collaboration—thicker lines represent stronger collaborative ties. **(B)** Comparison of the top 100 most-cited publications from China, the United States, and the European Union. **(C)** Comparative evaluation of the impact factors of the top 100 publications from China, the United States, and the European Union. *P < 0.05, **P < 0.01, ***P < 0.001, ****P < 0.0001.

Given the close collaboration among the European Union member states, the European Union in many aspects functions similarly to a federal state. Therefore, we treated the European Union as a single entity in our analysis. A publication threshold of six documents was applied to define active countries, resulting in the inclusion of the top 21 most productive countries for the interaction network analysis (**Figure 5A**).

The interaction map revealed that these top 21 countries could be grouped into three major clusters based on collaboration strength: Cluster 1: Led by the United States, the European Union, and the United Kingdom; Cluster 2: Led by China and Singapore; Cluster 3: Led by Japan and India. Notably, the lines connecting the United States, the European Union, and China were the thickest in the network, indicating the strongest international collaborations among them. Notably, the lines connecting the United States, the European Union, and China were the thickest in the network, indicating the strongest international collaborations among them.

A comparative analysis of total publication volume and citation performance (**Supplementary Figure S1**) showed that the United States (total citations: 74,989; normalized citations: 1,843.5), the European Union (37,486; 1,026.8), and China (45,666; 2,703.7) were the top 3 most influential contributors in the field. Although the European Union produced only 685 publications, its total citation count reached 37,486, resulting in an average citation per article of 54.7—lower than that of the United States (58.1) but significantly higher than China (14.7).

To further evaluate their academic influence, we analyzed the top 100 most-cited publications from the United States, China, and the European Union, based on the affiliation of the first author (see **Supplementary Table S2**). The composition of these top-cited papers varied slightly: the United States contributed 92 original research articles and 8 reviews; China contributed 87 articles and 13 reviews; and the European Union contributed 79 articles and 21 reviews. The analysis revealed that the United States had significantly higher citation counts for its top 100 papers compared to the European Union (*P* < 0.0001) and China (*P* < 0.001) (**Figure 5B**). Similarly, the impact factors of the journals in which these top papers were published were also significantly higher for the United States than for the European Union (*P* < 0.0001) and China (*P* < 0.001) (**Figure 5C**).

### Literature analysis

**Figure 6:**
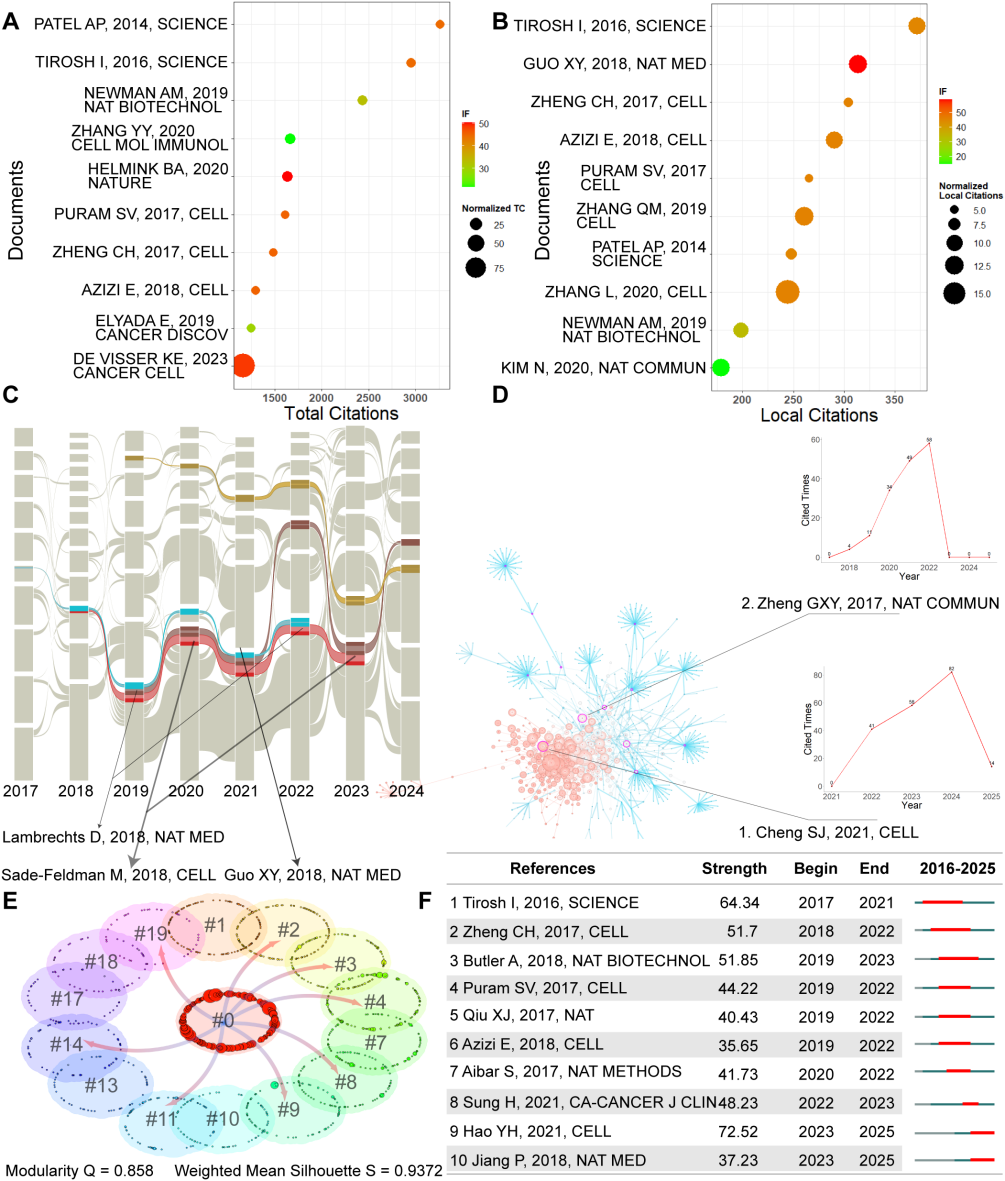
literature analysis. **(A)** Top 10 most globally cited publications. **(B)** Top 10 most locally cited publications. **(C)** Alluvial flow map of representative literature. **(D)** Literature co-citation analysis network. **(E)** Cluster analysis of co-cited literature. **(F)** Burst detection analysis of highly influential literature ranked by starting year.

**Figure 6A** presents the global top 10 most cited articles in this field. To control for the effects of publication year and journal subject category, both raw impact factors and normalized citation frequencies were included. As shown, the article by Patel et al. (2014, *Science*) received the highest total citation count. This landmark study pioneered single-cell sequencing in the context of tumor heterogeneity, revealing substantial transcriptional differences between individual cells within the same tumor and even within different bowel regions of the same patient (18). These findings underscore the critical need to account for intratumoral heterogeneity in the design of effective immunotherapies.

The review by De Visser et al. (2023, *Cancer Cell*) ranked highest in terms of journal impact factor and standardized citation frequency. This article provides comprehensive insights into the role of TME in cancer progression, emphasizing its dynamic and evolving nature as a determinant of tumor behavior (19).

**Figure 6B** highlights the top 10 most locally cited articles—those with the highest citation frequencies within the analyzed dataset. Among them, the study by Tirosh et al. (2016, *Science*) had the highest local citation count, representing the first systematic single-cell-level characterization of the multicellular ecosystem in malignant melanoma (20). Zhang et al. (2020, *Cell*) (21) achieved the highest normalized citation frequency, while Guo et al. (2018, *Nature Medicine*) (22) was published in the journal with the highest impact factor among the top local citations.

To avoid underestimating emerging high-impact studies that may not have yet accumulated sufficient citations due to their recent publication dates, we applied normalized citations as a screening criterion. Specifically, we identified the top 10 documents based on local normalized citation frequency and the top 10 based on global normalized citation frequency (**Table 2**). Notably, the majority of these papers were published after 2022, highlighting the growing influence of recent research in this field.

**Table 2:** Top 10 documents ranked by normalized citation impact.

Rank	Document	Normalized Local Citations	Document	Normalized Global Citations
1	YE BC, 2025, BIOFACTORS	142.00	**DE VISSER KE, 2023, CANCER CELL**	90.87
2	LI PH, 2025, CURR MED CHEM	142.00	HE SY, 2025, NAT BIOTECHNOL	81.14
3	ZHANG YY, 2025, CANCER CELL	142.00	SUN YF, 2025, HEPATOLOGY	50.71
4	LIN ZH, 2024, FRONT IMMUNOL	55.65	LAMARCHE NM, 2024, NATURE	21.89
5	XING J, 2024, FRONT IMMUNOL	48.23	YU YF, 2025, INT J SURG	20.29
6	**DE VISSER KE, 2023, CANCER CELL**	47.42	WU YC, 2022, CANCER DISCOV	19.57
7	SHAO WW, 2024, FRONT IMMUNOL	44.52	**LIU Y, 2023, J HEPATOL**	19.48
8	**LIU Y, 2023, J HEPATOL**	43.24	MA P, 2024, CIRCULATION	18.55
9	**MA JQ, 2024, SCIENCE**	40.81	**MA JQ, 2024, SCIENCE**	17.44
10	WU YC, 2022, CANCER DISCOV	38.05	YARCHOAN M, 2024, NAT MED	16.69

^#^Publications marked in bold are shared across both lists.

Our analysis revealed distinct thematic focuses between the local and global top 10 papers. The local top 10 papers predominantly concentrate on single-cell atlases of cancer [local top 1 (23), 3 (24), 4 (25), 5 (26), 7 (27)] and immune cell populations [local top 2 (28), 5, 8 (29), 9 (30), 10 (31)]. In contrast, the global top 10 papers place greater emphasis on predictive models [global top 2 (32), 5 (33)] and clinical applications [global top 10 (34)]. Of particular note, the shared local top 6 and top 8 publications provide in-depth insights into the TME (19, 29), while the shared top 9 paper presents a comprehensive cross-cancer atlas of B-cell populations (30).

Subsequently, we analyzed the top 10% of most frequently cited references from 1998 to 2025 using CiteSpace (**Figure 6C**), limiting the maximum number of selected references to no more than 100 per year. The resulting annual networks were then imported into the Alluvial Generator (https://www.mapequation.org/apps/MapGenerator.html) for visualization of citation flow and temporal evolution. For network modeling, a six-link strength threshold and a six-year backtracking window were applied for each node to trace persistent citation trajectories. The analysis revealed that citation flows lasting more than six years were predominantly concentrated between 2017 and 2024, indicating a sustained and intensifying scholarly focus during this period.

**Figure 6C** highlights several pivotal cross-linking nodes situated at the intersection of major citation flows, suggesting their central role in shaping the field. Notable examples include *Lambrechts D., 2018, Nature Medicine* (35); *Sade-Feldman M., 2018, Cell* (36); and *Guo X.Y., 2018, Nature Medicine* (22)—all of which represent foundational contributions to the development of single-cell and immunotherapy research.

Furthermore, a co-citation analysis was performed on the top 10% most-cited references from 2017 to 2025, with a maximum of 100 references included per year (**Figure 6D**). The analysis identified two key publications—referred to as Article 1 and Article 2—with notably high betweenness centrality, indicating their pivotal role in connecting different research clusters within the field. The temporal trends in their local citation frequencies are illustrated in the accompanying inset graphs. Article 1, *Cheng S.J., 2021, Cell*, has steadily gained attention for its comprehensive exploration of the composition and functional heterogeneity of tumor-infiltrating myeloid cells across various cancer types. The study underscores the importance of accounting for intertumoral differences in myeloid cell profiles when designing targeted immunotherapeutic strategies (7). Article 2, *Zheng G.X.Y., 2017, Nature Communications*, exhibited a burst strength of 21.6816 and began to attract widespread interest starting in 2018, with sustained influence through 2022. This study introduces a high-throughput single-cell RNA sequencing platform based on microdroplet technology—namely, 10x Genomics’ GemCode system. This technological breakthrough represents a significant milestone in scalable single-cell transcriptomic profiling. Its application has not only advanced our understanding of the tumor immune microenvironment but also laid a critical foundation for the development of novel immunotherapeutic approaches (37).

To further explore the intellectual structure of single-cell research in immunotherapy from 1998 to 2025, a co-citation analysis was conducted using the g-index algorithm (k = 25) to identify highly influential references. To delineate the longitudinal evolution of research themes, a clustering analysis was performed based on article titles as indexing terms. Cluster formation and thematic labeling were carried out using a combination of the llama3 8B language model, Latent Log-Likelihood Ratio (LLR), and Semantic Indexing (LSI) methods (**Figure 6E**). The detailed naming and characteristics of each cluster are provided in **Supplementary Table S3**.

As shown in **Figure 6E**, cluster 0 appears to represent an emergent and evolving core theme within the field, likely originating from clusters 2, 3, 4, 8, 9, 11, 14, and 19—indicating a convergent trajectory in research focus. Cluster 0 is characterized by studies centered on the tumor immune microenvironment, a key concept in understanding immunotherapeutic mechanisms at the single-cell level. Representative publications within this cluster include seminal works such as Tirosh et al. (2016, *Science*) (20), which introduced single-cell transcriptomic profiling in melanoma; Newman et al. (2019, *Nature Biotechnology*) (38), who developed CIBERSORTx for cell-type deconvolution; Helmink et al. (2020, *Nature*) (39), which investigated tertiary lymphoid structures and immunotherapy response; and Puram et al. (2017, *Cell*) (40), which mapped head and neck squamous cell carcinoma at single-cell resolution.

The originating clusters that feed into #0 reflect a diverse but interconnected research landscape: Mass Cytometry (#2), Tumor Immune Signatures (#3), Cancer Immune Dynamics (#4), Immunotherapy Response Prediction (#8), Precision Medicine (#9), High-Throughput Single-Cell Microtechnologies (#10), T Cell Dysfunction (#11), Functional Proteomics (#14) and Cancer Immune Cell Dynamics (#19).

This thematic convergence underscores the increasing integration of high-dimensional single-cell technologies, computational modeling, and systems immunology to decode the spatial, phenotypic, and functional complexity of the tumor-immune interface.

Subsequently, the top 10 references with the strongest citation bursts were identified and visualized in **Figure 6F**. Among these, *Tirosh I, 2016, Science* (20) exhibited the highest burst intensity (strength = 73.47), with sustained high attention from 2017 to 2021. This pivotal study leveraged single-cell transcriptomic techniques to dissect the cellular composition and functional states within metastatic melanoma, highlighting the profound complexity of the tumor and its microenvironment. Following closely is *Zheng C.H., 2017, Cell*, which demonstrated a burst strength of 55.82. This study utilized single-cell transcriptomic analysis to reveal the heterogeneity of dendritic cells and monocytes in human peripheral blood, leading to the identification of novel immune cell subsets (6). These findings have opened new avenues for understanding immune system dynamics and designing immunotherapeutic strategies.

### Keyword analysis

**Figure 7:**
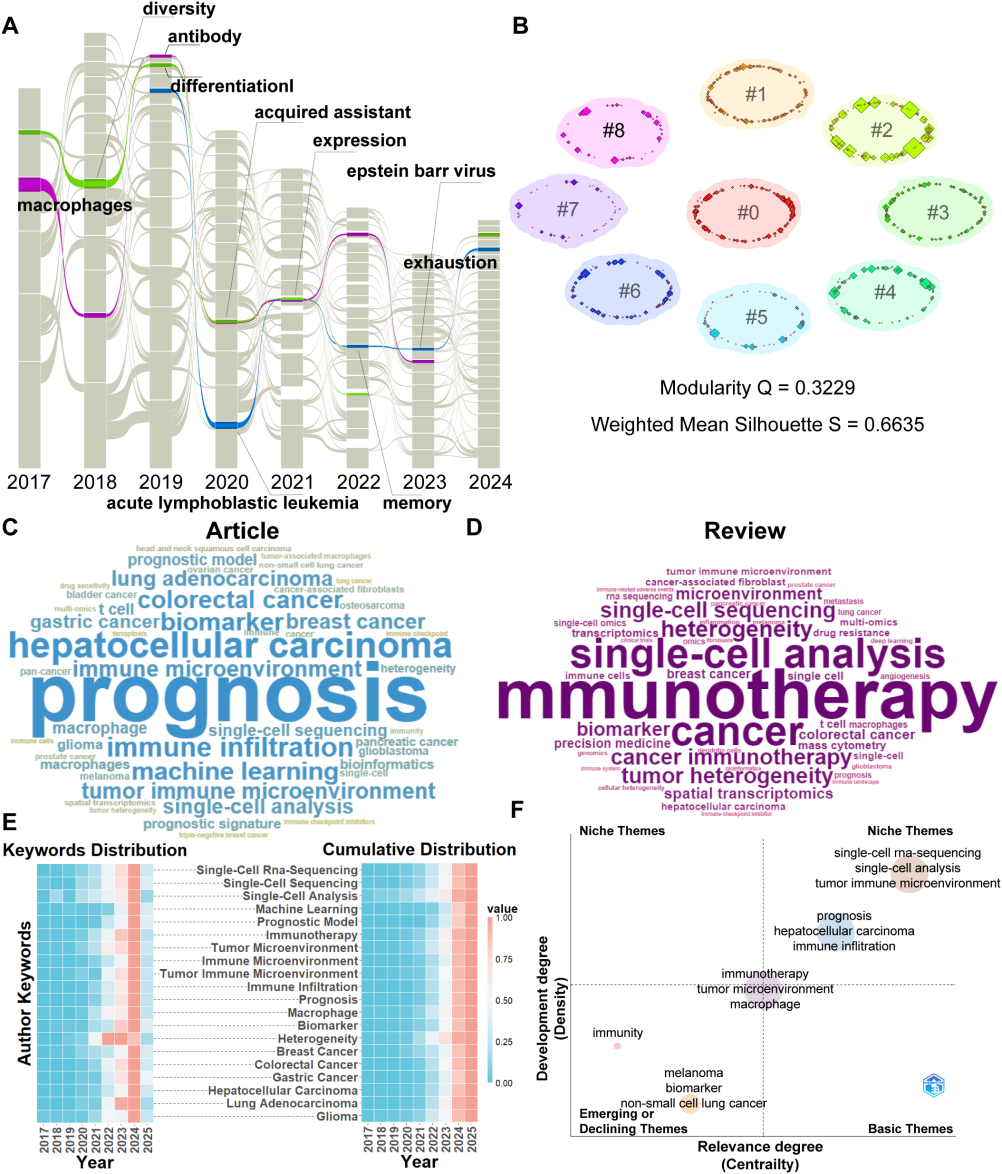
Keyword analysis. **(A)** Alluvial flow map of keywords. **(B)** Cluster analysis based on keyword co-occurrence. **(C)** Word cloud of author keywords in research articles. **(D)** Word cloud of author keywords in review articles. **(E)** Heatmap of the top 20 most frequent author keywords. **(F)** Strategic diagram (strategic coordinate map) of keyword clusters.

To capture a broader range of keywords, the g-index algorithm (k = 25) in CiteSpace was utilized (**Figure 7A**). After exporting the.net file, the data were imported into the MapEquation platform (https://www.mapequation.org/apps/MapGenerator.html) to generate an impact flow diagram. For network modeling, each node was assigned six of its strongest connections, and a backtracking window of six years was applied. The analysis identified three persistent thematic streams, each extending for more than six years and concentrated within the period from 2017 to 2024—consistent with the temporal pattern observed in the reference citation flow. Representative keywords in these streams are annotated in the figure. Notably, cross-cutting terms such as “acquired resistance” and “expression” suggest that researchers have maintained long-term interest in understanding resistance mechanisms and gene expression dynamics in the context of single-cell analysis of cancer immunotherapy. Based on the dominant keywords within the three major streams, the long-standing research foci since 2017 can be broadly categorized into the following areas: Heterogeneity and differentiation of resistant tumor cells – represented by keywords such as *diversity* and *differentiation*, highlighting the evolving landscape of tumor cell plasticity under therapeutic pressure; Immune resistance induced by antibody-based therapies – mediated by *macrophages* and *antibodies*, reflecting research on how therapeutic antibodies can unintentionally drive immune cell dysfunction or tolerance; Immune cell exhaustion and memory impairment – exemplified by terms such as *acute lymphoblastic leukemia*, *memory*, *Epstein-Barr virus*, and *exhaustion*. This reflects interest in T cell exhaustion, wherein chronic antigen exposure and inflammation impair effector functions and memory formation, particularly in cancer and persistent viral infections.

Furthermore, a keyword co-occurrence clustering analysis was performed using CiteSpace (**Figure 7B**), yielding a modularity value of Q = 0.3229 and a silhouette score of S = 0.6635, indicative of a moderately robust and internally consistent clustering structure. Cluster labeling was facilitated through integration of the llama 3B language model, in combination with LLR and LSI algorithms to improve thematic interpretability. Detailed cluster descriptors and characteristics are provided in **Supplementary Table S4**. The analysis identified several major thematic domains within the field, with the largest and most central cluster labeled as #0: Cancer Microenvironment Dynamics. Other prominent clusters include: #1 Single-Cell Analysis; #2 Tumor; Microenvironment; #3 Disease Diagnosis Tools; #4 Immune Cell Dynamics; #5 Tumor Microenvironment Analysis; #6 Machine Learning; #7 Receptor; #8 Cancer Immunotherapy.

To further investigate research hotspots, articles (**Figure 7C**) and reviews (**Figure 7D**) were analyzed separately using word cloud analysis based on authors’ keywords. For articles, the most frequently occurring bioinformatics-related keywords included: *single-cell RNA sequencing* (836), *machine learning* (142), *single-cell analysis* (118), *single-cell sequencing* (94), *prognostic model* (87), *bioinformatics* (77), *spatial transcriptomics* (47), and *multi-omics* (42). The most commonly studied cancer types were: *hepatocellular carcinoma* (208), *colorectal cancer* (141), *breast cancer* (121), *lung adenocarcinoma* (121), *gastric cancer* (108), *pancreatic cancer* (68), *glioblastoma* (64), *melanoma* (57), *bladder cancer* (56), *osteosarcoma* (54), *pan-cancer* (54), *ovarian cancer* (53), *prostate cancer* (46), *squamous cell carcinoma* (45), and *triple-negative breast cancer* (36).

In terms of cancer treatment and immune-related research, frequently cited terms included: *tumor microenvironment* (618), *immunotherapy* (600), *prognosis* (470), *immune infiltration* (155), *immune microenvironment* (127), *tumor immune microenvironment* (125), *biomarker* (144), *macrophage* (90), *prognostic signature* (78), *heterogeneity* (64), *cancer-associated fibroblasts* (52), *tumor-associated macrophages* (41), *tumor heterogeneity* (39), *drug sensitivity* (38), *ferroptosis* (37), *immune checkpoint* (37), and *immune checkpoint inhibitors* (36).

For reviews, the keyword frequency was generally lower, but several specific themes emerged. Frequently mentioned bioinformatics-related terms included *deep learning* (5) and *mass cytometry* (9). Keywords related to cancer treatment and immunological mechanisms included: *heterogeneity* (18), *tumor heterogeneity* (17), *precision medicine* (10), *drug resistance* (8), *angiogenesis* (6), *immune-related adverse events* (4), and *immune landscape* (4).

These findings reflect a growing emphasis on computational methods, immune interactions, and tumor heterogeneity in both original research and comprehensive reviews within the field of single-cell immunotherapy.

To further illustrate the temporal evolution and emerging trends of research topics, a heatmap analysis was conducted based on the annual frequency of the top 20 author keywords (**Figure 7E**). The results show that keywords such as *machine learning*, *prognostic model*, *immune microenvironment*, and heterogeneity have demonstrated a clear upward trend in recent years, suggesting that these topics are gaining increasing attention and are likely to remain focal points in future research. Keyword relevance analysis revealed that the tumor immune microenvironment is positively associated with tumors, while the tumor microenvironment shows a positive correlation with spatial transcriptomics. (**Supplementary Figure S2**).

To evaluate the developmental stage and research maturity of various topics in the field of single-cell immunotherapy, a thematic strategic diagram was constructed (**Figure 7F**). This strategic coordinate plot categorizes topics based on centrality (representing importance and connectivity to other themes) and density (indicating the degree of internal development).

Core foundational topics such as *single-cell RNA sequencing*, *single-cell analysis*, and *tumor immune microenvironment* occupy the lower-right quadrant, indicating high centrality but relatively low density—suggesting they are well-established and broadly connected to other topics but still offer significant potential for further in-depth exploration.

Topics such as *immunotherapy*, *tumor microenvironment*, and *macrophage* appear in the upper-middle quadrant. These themes possess relatively high centrality and moderate density, indicating they serve as hubs that connect multiple research directions and have already undergone considerable investigation. However, their moderate internal cohesion also highlights the opportunity for further refinement and specialization.

Collectively, these topics form the core framework of “broadly connected and expandable” research within the field and represent valuable entry points for deepening and integrating future interdisciplinary efforts.

### Journal analysis

**Figure 8:**
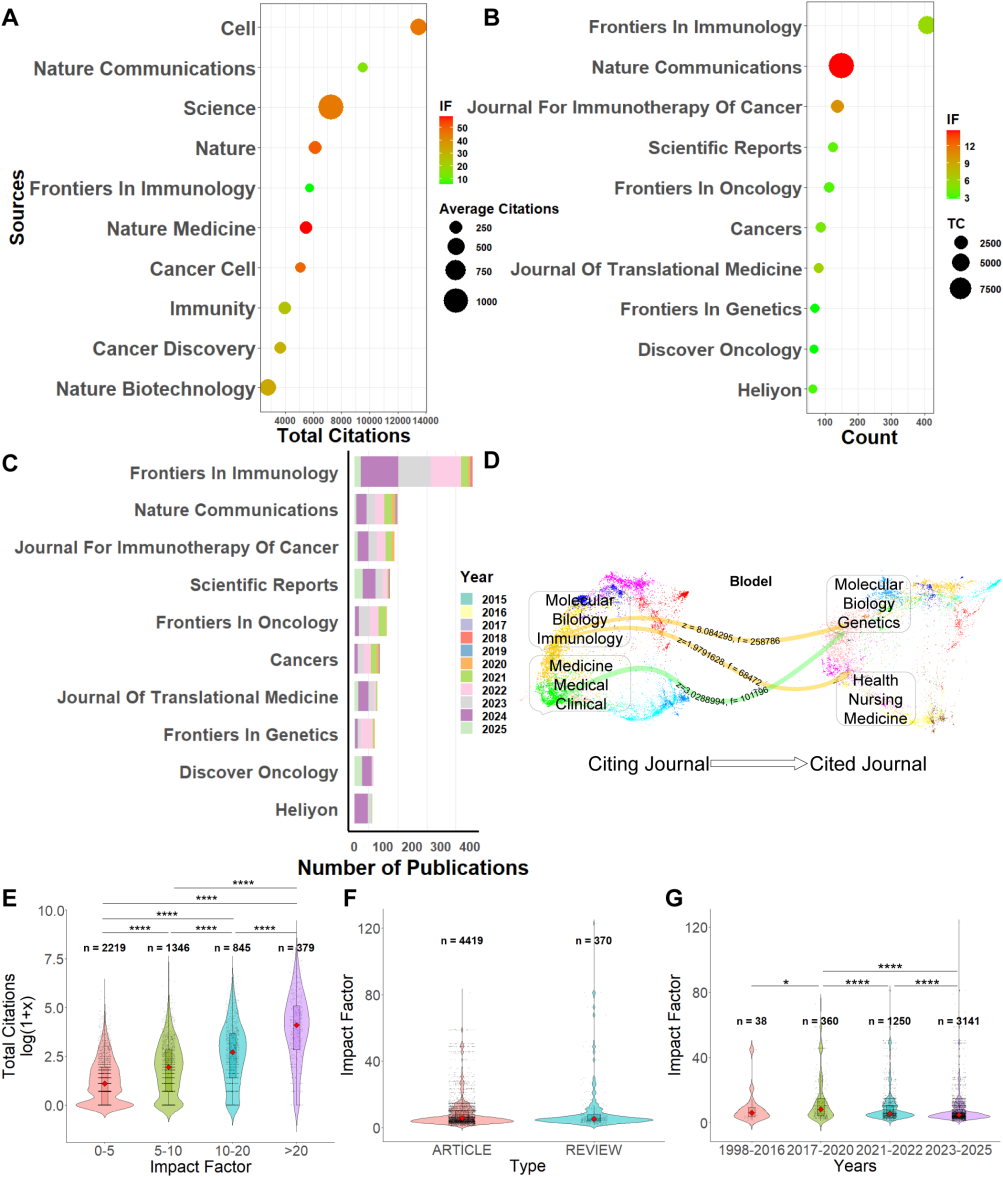
Journal analysis. **(A)** Top 10 journals ranked by total citation count. **(B)** A Top 10 journals ranked by number of publications. **(C)** Annual publication trends of the top 10 journals. **(D)** Dual-map overlay of journal citation relationships. **(E)** Distribution of total citations across different journal impact factor ranges. **(F)** Impact factor comparison across different article types. **(G)** Distribution of journal impact factors across different publication years. *P < 0.05, **P < 0.01, ***P < 0.001, ****P < 0.0001.

A bubble chart was generated to visualize the top 10 most-cited journals, illustrating their total citation counts, number of publications, average citations per article, and impact factors (**Figure 8A**). The top 5 most frequently cited journals are *Cell* (13491), *Nature Communications* (9482), *Science* (7272), *Nature* (6130), and *Frontiers in Immunology* (5713). Among them, *Science* exhibits the highest average citation per article (1038.9), followed by *Cell* (465.2). In terms of impact factor, *Nature Medicine* (58.7) ranks first, followed by *Nature* (50.5).

Subsequently, a bubble chart of the top 10 journals with the highest number of publications was constructed (**Figure 8B**). The leading three journals in terms of publication volume are *Frontiers in Immunology* (410), *Nature Communications* (150), and *Journal for Immunotherapy of Cancer* (138). *Nature Communications* (14.7) ranks highest in both total citation frequency and impact factor, followed closely by *Frontiers in Immunology* (5.7).

Further temporal analysis based on publication year (**Figure 8C**) revealed that the peak period of publication activity occurred between 2022 and 2024. *Frontiers in Immunology* maintained the highest annual publication volume throughout this period, followed by *Nature Communications*.

The superposition of dual-map overlays was used to illustrate the knowledge flow paths (**Figure 8D**). As shown in the figure, research from the molecular, biological, and genomics fields, along with health and medical sciences, converges into the molecular, biological, and immunological disciplines. Furthermore, molecular, biological, and genomic studies have extended into clinical, pharmaceutical, and medical fields.

To explore the relationship between journal impact factors and citation frequency, impact factors were divided into four categories: 0–5, 5–10, 10–20, and >20. As shown in **Figure 8E**, articles published in journals with an impact factor greater than 20 had significantly higher citation frequencies compared to those in the 10–20 range (p < 0.0001). Similarly, articles in the 10–20 impact factor range were cited more frequently than those in the 5–10 range (p < 0.0001), and articles in the 5–10 range had higher citation frequencies than those with impact factors between 0–5 (p < 0.0001).

Differences in impact factors between original research articles and review articles were also assessed (**Figure 8F**), revealing no statistically significant difference. Additionally, the temporal distribution of impact factors was analyzed (**Figure 8G**). Publications from 2017 to 2020 had significantly higher impact factors than those from 1998 to 2016 (p = 0.01). Furthermore, impact factors in 2021–2022 were significantly greater than those in 2017–2020 (p < 0.0001), and impact factors in 2023–2025 surpassed those of both 2017–2020 and 2021–2022 (p < 0.0001 for both comparisons).

## Discussion

### Basic overview of the industry

The field of single-cell analysis in tumor immunotherapy has undergone substantial development since the late 20th century. Publication frequency serves as a key indicator for assessing the emergence and evolution of a research hotspot. During its nascent stage, this area garnered limited attention, with fewer than eight articles published annually. A notable turning point occurred in 2016, when researchers began to recognize the potential of single-cell technologies in advancing cancer immunotherapy. This recognition spurred a rapid increase in research activity and scientific output. From 1998 to 2015, publication numbers remained relatively stable with minimal growth. However, between 2016 and 2019, the annual growth rate rose significantly to 27.86%. This upward trajectory accelerated dramatically between 2020 and 2024, reaching an impressive growth rate of 294.18%.

Differences in authorship and citation patterns provide further insights into collaborative practices and the integration of knowledge within this emerging field. The higher number of authors in original research articles likely reflects the multidisciplinary and collaborative nature of experimental work. In contrast, the greater number of references cited in review articles underscores their role in synthesizing rapidly expanding findings to inform clinical and translational applications.

It is important to note that older publications naturally tend to accrue more citations over time, potentially biasing impact assessments. Therefore, incorporating additional metrics—such as normalized citation frequency and citation burst analysis—offers a more comprehensive and time-adjusted evaluation of scientific influence. These approaches help ensure that recently published high-impact studies are appropriately recognized, despite their limited time to accumulate citations.

The top seven countries contributing to the literature in this field are China, the United States, Germany, the United Kingdom, Japan, South Africa, and France. Among them, the United States, China, and the European Union exhibit the highest total citation counts, publication outputs, and normalized citation frequencies. Notably, the top 100 most-cited publications from the United States have significantly higher citation frequencies and journal impact factors compared to those from the European Union and China, indicating a stronger academic influence and higher international visibility. Although China leads in publication volume, its average citations per article are significantly lower than those of the United States and the European Union, suggesting relatively limited international impact and recognition.

The five institutions with the highest publication frequencies in this field are Fudan University, Sun Yat-sen University, Shanghai Jiao Tong University, Nanjing Medical University, and Central South University. Given current trends, it is anticipated that scholarly output in this area will continue to grow in the coming years. This growth is likely to be accompanied by increasingly close international collaboration, reflecting the global importance and interdisciplinary nature of single-cell analysis in tumor immunotherapy.

Journal analysis highlights the highly interdisciplinary nature of this field, with citation sources primarily originating from foundational biology and tumor immunology journals such as *Cell*, *Cancer Cell*, and *Nature Immunology*. In contrast, knowledge output is mainly concentrated in journals positioned at the intersection of oncology and immunotherapy, including the *Journal for Immunotherapy of Cancer* and *Clinical Cancer Research*.

The strategic diagram further illustrates that topics such as “tumor microenvironment heterogeneity” and “CD8^+^ T cell exhaustion” exhibit both high centrality and high density, indicating they are motor themes—well-developed and influential research areas driving the field forward. In contrast, “multimodal single-cell omics integration” occupies the quadrant of high centrality but low density, suggesting it is an emerging or transversal theme with substantial potential for future development.

### Mainstreaming research directions

According to keyword analysis, research in this field is primarily driven by terms such as *single-cell RNA sequencing*, *machine learning*, *prognostic models*, *bioinformatics*, *spatial transcriptomics*, *multi-omics*, *tumor microenvironment*, *immune infiltration*, *immune microenvironment*, *heterogeneity*, *macrophage*, *drug sensitivity*, *ferroptosis*, and *immune checkpoints*. Based on our keyword and co-citation clustering analyses, the major research directions can be classified into the following categories:

The frequent appearance of keywords such as *immune microenvironment*, *tumor immune microenvironment*, *heterogeneity*, and *tumor heterogeneity* suggests that immune cell subsets within tumors exhibit considerable heterogeneity. This makes it challenging to identify key cell populations through bulk sequencing techniques. The emergence of *spatial transcriptomics* offers a promising approach to dissect the spatial organization of TME, highlighting its growing relevance in this area. The results of the keyword relevance analysis further validate their association. Representative studies in this cluster, such as Lavin et al. (*Cell*, 2017) (41) and Li et al. (*Frontiers in Immunology*, 2022) (42), focus on the spatial distribution and functional dynamics of immune cells—including T cells, NK cells, and myeloid cells—across tumor tissues, adjacent normal tissues, and peripheral blood. These studies leverage single-cell transcriptomic technologies to construct immune atlases that reflect the evolutionary dynamics of the TME (23).

The recurrence of keywords such as *immunotherapy*, *cancer immunotherapy*, *immune checkpoints*, and *immune checkpoint inhibitors* highlights the central role of immune checkpoints in therapeutic strategies. Additionally, the frequent mention of *drug sensitivity* reflects growing attention to immune resistance mechanisms. This cluster includes studies exploring the synergistic effects of combining PD-1 with GITR-targeted immunotherapy to restore CD8^+^ T cell functionality and maintain memory phenotypes, underscoring the potential of combinatorial approaches to enhance anti-tumor immunity.

This category encompasses the technological evolution of single-cell sequencing platforms, which can be divided into four distinct phases.

Germination Stage (pre-2009): Early efforts focused on adapting high-throughput sequencing to single-cell analysis. However, limitations such as low throughput, high amplification bias, and poor reproducibility hindered progress. The breakthrough came in 2009 with the first successful single-cell mRNA sequencing, marking the inception of modern single-cell transcriptomics (43). These early methods laid the foundation for profiling immune cell diversity within tumors, which is essential for understanding the mechanisms of immune evasion. Rapid Development Stage (2010–2015): A foundational technological framework was established, with advances in cell capture, amplification, library construction, and data analysis. Several widely used platforms emerged during this period, such as Fluidigm C1 and SMART-seq (44–46), enabling the first studies that revealed the heterogeneity of tumor-infiltrating lymphocytes and identified early biomarkers of immunotherapy response. Maturation and Diversification Stage (2016–2019): This period was marked by the expansion of multi-omics capabilities and the profiling of diverse cell types. Notable milestones include the introduction of the 10x Chromium platform by 10x Genomics (Zheng et al., 2017), which set a new industry standard (37), and the development of Seurat v3 by Stuart et al. (2019), which facilitated data integration across single-cell studies (47). These advancements enabled large-scale mapping of immune cell states, such as exhausted T cells, contributing to the refinement of immune checkpoint blockade strategies. Spatial transcriptomics, pioneered by Stahl et al. in 2016 (48), further allowed researchers to visualize immune cell interactions in TME. Refinement and Clinical Translation Stage (2020–present): This phase emphasizes higher resolution, multidimensional analysis, and clinical applicability. For example, Penghui Li et al. conducted single-cell analyses of esophageal cancer and adjacent tissues, discovering that a high proportion of HSPA6^+^ macrophages is negatively correlated with patient survival outcomes (28). Yuanyuan Zhang et al. performed a comparative single-cell analysis of immune cell dynamics in triple-negative breast cancer patients treated with paclitaxel, nab-paclitaxel, and their combinations with the anti-PD-L1 antibody atezolizumab, providing a detailed view of immune cell transitions during immunotherapy (24). Jiaqiang Ma et al. integrated data from 269 patients across 20 cancer types to construct a comprehensive cross-cancer single-cell atlas of tumor-infiltrating B cells, providing a valuable resource for identifying therapeutic targets and biomarkers (39). Importantly, these single-cell studies have directly shaped the landscape of cancer immunotherapy by identifying tumor-reactive T cell subsets, uncovering predictive markers for PD-1 blockade response, and informing the development of personalized cancer vaccines.

### Single-cell technology pushes immunotherapy to be precise and individualized

The substantial heterogeneity of immune cells within TME remains a major barrier to achieving consistent and durable responses to immunotherapy. Traditional bulk sequencing approaches often mask this complexity, whereas scRNA-seq enables the identification of diverse immune subpopulations—including CD8^+^ T cells, regulatory T cells (Tregs), M1/M2 macrophages, and exhausted T cells—revealing their relative abundances and functional states across individual patients. For example, Sade-Feldman et al. utilized scRNA-seq to profile tumor-infiltrating T cells in melanoma patients treated with immune checkpoint inhibitors. Their findings revealed that responders were enriched for activated CD8^+^ T cell phenotypes, whereas non-responders exhibited transcriptional signatures of T cell exhaustion (36). These insights underscore the potential of constructing immune cell–based “sensitivity maps” to guide personalized therapeutic strategies. Notably, the recent construction of cross-cancer single-cell atlases of infiltrating B cells has attracted widespread attention, further emphasizing the importance of this approach (30).

Beyond profiling canonical checkpoint molecules such as PD-1 and CTLA-4, scRNA-seq facilitates the discovery of novel immunoregulatory pathways and inhibitory receptors. Emerging checkpoint targets—such as LAG3, TIM3, and TIGIT—have been identified in dysfunctional T cell subsets within TME and are increasingly recognized as drivers of immune escape (49). These findings have provided a strong rationale for combinatorial immune checkpoint blockade strategies, including dual targeting approaches (e.g., PD-1 plus LAG3). Furthermore, pathway-level analyses at the single-cell resolution reveal complex immunosuppressive cytokine networks and macrophage polarization imbalances, expanding the scope of potential therapeutic targets beyond traditional immune checkpoints.

By enabling high-resolution profiling of inter-individual variability in immune cell composition and functional states, single-cell technologies significantly advance the personalization of immunotherapy. Several studies have developed immune-related risk scores and T cell functional signatures using scRNA-seq data, demonstrating their predictive value for treatment response (50). Additionally, integrating scRNA-seq with peripheral blood analyses offers a minimally invasive approach to monitor dynamic immune changes, predict treatment outcomes, and detect potential immune-related adverse events in real time. Moreover, novel plasmablast-like cell signatures developed through the integration of single-cell omics and machine learning have demonstrated remarkable predictive power for immunotherapy efficacy, further demonstrating the potential of single-cell technology for prognostic evaluation of immunotherapy (23).

### Future research directions and prospects

Despite substantial progress, several critical challenges remain in the application of single-cell technologies to immunotherapy research. Future advancements should focus on the following key areas to fully harness the potential of these tools.

Tumor–immune interactions are orchestrated across multiple regulatory layers, including transcriptional, epigenetic, and proteomic mechanisms. Reliance on a single modality, such as scRNA-seq, often yields an incomplete picture of these complex dynamics. Future research should emphasize the integration of scRNA-seq with complementary technologies such as scATAC-seq, CITE-seq (for surface protein profiling), and spatial transcriptomics to construct multidimensional immune atlases (47). These integrative approaches can illuminate cell–cell communication pathways, immunosuppressive networks, and the spatial organization of immune subsets within the tumor microenvironment, thereby informing the rational design of synergistic immunotherapeutic strategies.

The majority of current single-cell studies rely on cross-sectional, single-time-point samples, which limit insights into the temporal dynamics of immune responses. Longitudinal sampling—before, during, and after therapy—will be crucial for tracking immune cell plasticity, clonal evolution, and mechanisms of immune escape over time (51). Such dynamic profiling will enable a deeper understanding of resistance pathways and facilitate timely therapeutic adaptations based on real-time immune trajectories.

The complexity and high dimensionality of single-cell datasets pose significant analytical challenges. Artificial intelligence (AI), particularly deep learning algorithms, holds great promise for enhancing cell-type annotation, trajectory inference, and response prediction. Integrating AI into single-cell data analysis pipelines could dramatically improve interpretability, enable robust prognostic modeling, and accelerate the development of clinical decision support systems tailored to individual patients. A compelling example of the integration between single-cell omics and AI is the recent development of deep learning–based multimodal models that combine pathology images with genomic and transcriptomic data to predict preoperative lymph node specificity and disease-free survival, illustrating the translational potential of AI-augmented single-cell analysis in precision oncology (33).

To date, many findings from single-cell studies are derived from small cohorts or retrospective analyses. To ensure clinical relevance, future research must prioritize large-scale, prospective, multi-center validation studies that encompass diverse cancer types and patient populations. Rigorous validation is essential for transforming single-cell–based discoveries into actionable biomarkers and therapeutic targets with real-world clinical applicability.

In conclusion, single-cell technologies have become indispensable tools for unraveling the intricacies of the tumor immune microenvironment and refining immunotherapeutic approaches. Continued progress will depend on the integration of longitudinal immune monitoring, multi-omics profiling, AI-enhanced analytics, and robust clinical validation. Together, these efforts will bridge the gap between single-cell research and precision oncology, ultimately contributing to more effective, individualized cancer immunotherapies.

### Strengths and limitations

In this study, we present the first systematic and visually intuitive bibliometric analysis of publications and emerging trends concerning the application of single-cell analysis in cancer immunotherapy. By employing a suite of bibliometric tools, including CiteSpace, VOSviewer, and Bibliometrix, we delineate the evolution of research hotspots, identify influential authors and institutions, and map out potential future directions in this rapidly advancing field. Our findings provide scholars and clinicians with a structured understanding of the current knowledge landscape and offer novel insights to guide subsequent investigations.

Nevertheless, several limitations should be acknowledged. First, due to the technical constraints of existing bibliometric platforms, we were unable to integrate data across multiple databases such as Scopus, PubMed, CNKI, and patent repositories in both Chinese and English. Consequently, our analysis was limited to WOSCC, which, while high in quality and comprehensiveness, does not fully capture all relevant literature. Future studies could benefit from more robust and integrative platforms capable of incorporating multi-source bibliographic data. Second, although meticulous search strategies were employed, the retrieval process inevitably included some irrelevant publications and may have omitted others due to ambiguous, inconsistent, or non-standardized titles and keywords. This highlights the inherent trade-off between sensitivity and specificity in bibliometric data collection. Third, the use of multiple bibliometric tools—each with different algorithms and visualization frameworks—may lead to slight variations in analytical outcomes. As with all quantitative analyses, these methodological differences, combined with inherent biases in citation behaviors, may influence the interpretation of results. Fourth, in this study, we used the 2023 JCR impact factor to represent journal influence, rather than the impact factor corresponding to the year of publication. This may limit the temporal accuracy of journal impact assessment. Future studies could consider incorporating year-specific impact factors or normalized citation metrics to allow for a more time-sensitive evaluation. Lastly, although we employed meticulous and refined search strategies, the retrieval process may have inevitably included irrelevant publications and omitted some relevant ones due to ambiguous, inconsistent, or non-standardized titles and keywords. This reflects the inherent trade-off between sensitivity and specificity in bibliometric data collection.

Despite these limitations, our study provides a timely and valuable overview of the scientific dynamics in single-cell–based cancer immunotherapy. With the continuous evolution of bibliometric methodologies and the expansion of relevant databases, future bibliometric analyses will further enhance our understanding of this field, facilitating the identification of cutting-edge themes and promoting interdisciplinary collaboration.

## Conclusion

This study offers the first comprehensive and scientifically grounded bibliometric overview of global research trends related to the application of single-cell analysis in cancer immunotherapy. By mapping the intellectual landscape and identifying emerging hotspots, our findings provide researchers with a clearer understanding of the field’s evolution and valuable guidance for future innovation. As AI continues to advance, the interdisciplinary integration of AI with multi-omics data is expected to catalyze the development of medical informatics. This convergence holds great promise for unraveling the mechanistic underpinnings of cancer immunotherapy and enhancing the precision of omics-driven strategies. Looking forward, the acceleration of integration between medicine and informatics will be critical. Priority should be given to strengthening links between clinical application, basic research, technological development, and standardization. Such efforts will support the modernization of medical informatics and promote translational outcomes. Within the framework of China’s “Belt and Road” Initiative and other favorable policy environments, enhanced international and interdisciplinary collaboration among researchers, institutions, and countries is anticipated. These synergies will foster deeper, more systematic investigations and ultimately contribute to delivering precise and personalized clinical guidance for cancer immunotherapy.

## Data Availability

The original contributions presented in the study are included in the article/**Supplementary Material**, further inquiries can be directed to the corresponding author/s.

## Supplementary material

Supplementary Table 1 Characteristic information form of all included papers.

Supplementary Figure 1 Analysis of total citation frequency, publication volume and normalized citation frequency of top 21 highly cited countries.

Supplementary Table 2 Top 100 most-cited publications from the United States, China, and the European Union.

Supplementary Table 3 Citation cluster naming results from the literature co-citation analysis.

Supplementary Table 4 Keyword co-citation cluster naming results.

Supplementary Figure 2 Results of the relevance analysis for key author keywords.
