# Supplementary figures and images for: Exploring research trends in cancer immunotherapy via single-cell technologies: a scientometric perspective
Source: Front Immunol. 2025 Aug 22;16:1640224. doi: 10.3389/fimmu.2025.1640224 (PMC12411161; doi:10.3389/fimmu.2025.1640224)

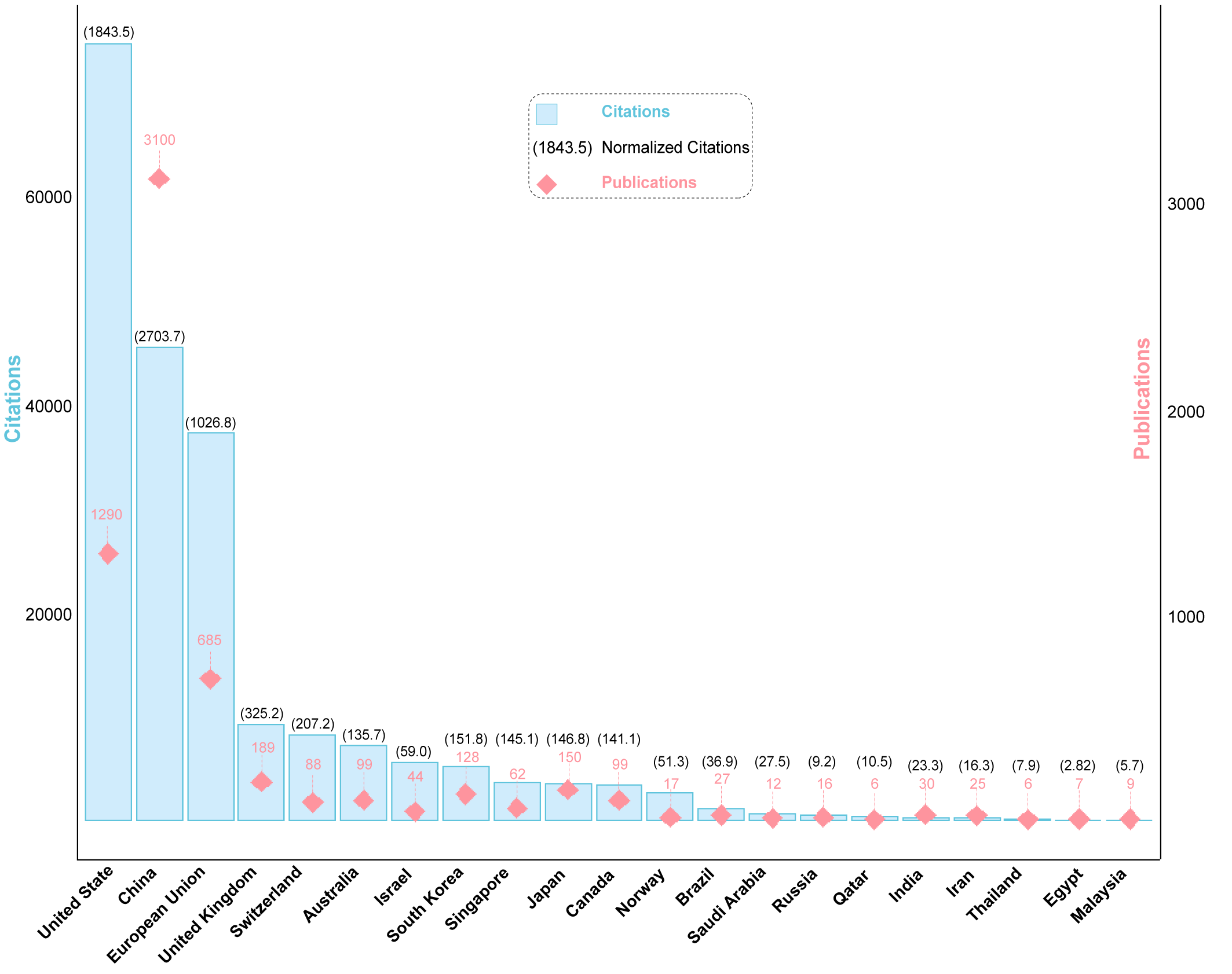

Supplement: Supplementary Figure 1 — Analysis of total citation frequency, publication volume and normalized citation frequency of top 21 highly cited countries. [file DataSheet2.zip › Additional file2/Figure S1.tif]

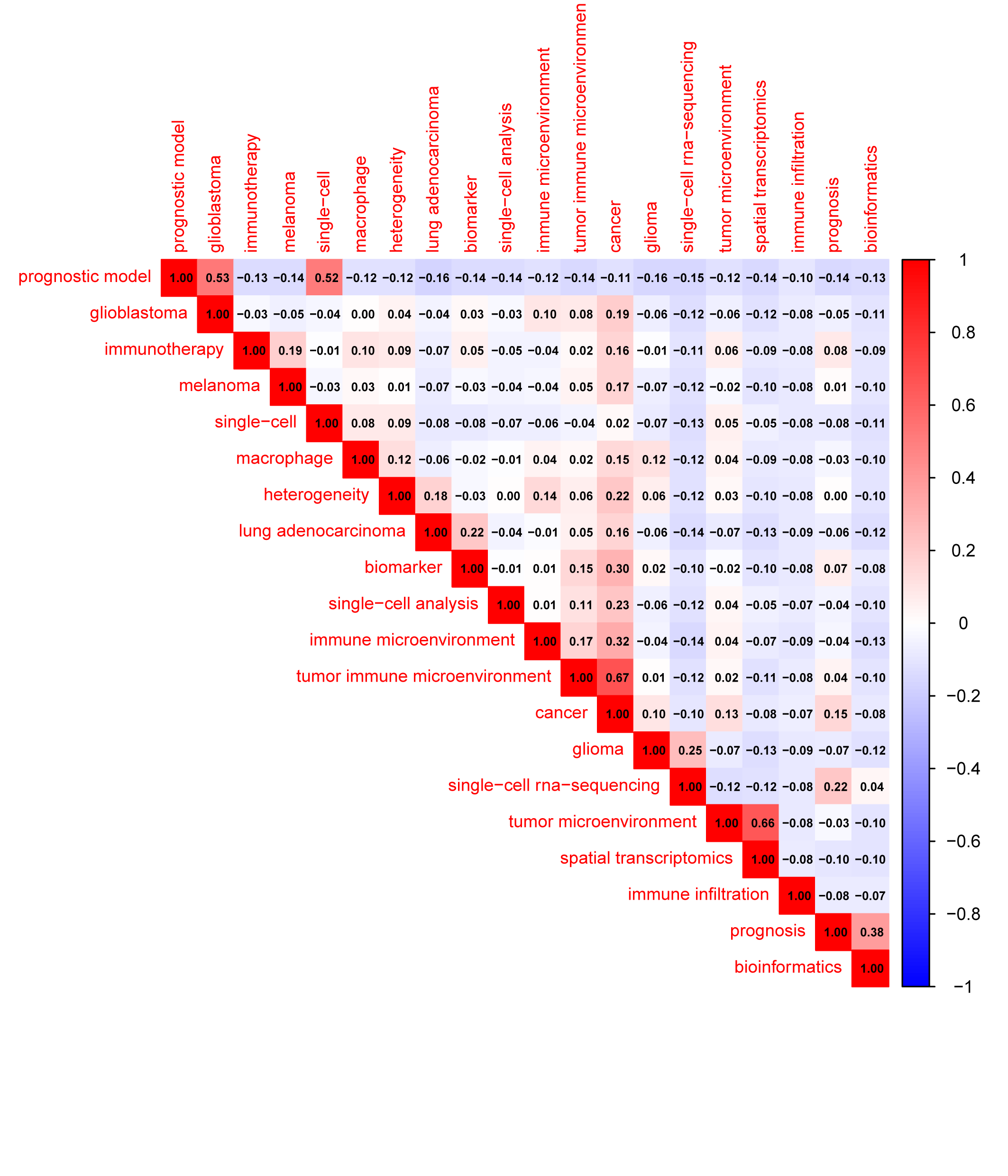

Supplement: Supplementary Figure 2 — Results of the relevance analysis for key author keywords. [file DataSheet6.zip › Additional file6/Figure S2.tif]
