# Supplementary material for: Exploring research trends in cancer immunotherapy via single-cell technologies: a scientometric perspective
Source: Front Immunol. 2025 Aug 22;16:1640224. doi: 10.3389/fimmu.2025.1640224 (PMC12411161; doi:10.3389/fimmu.2025.1640224)
Supplement: Supplementary Table 3 — Citation cluster naming results from the literature co-citation analysis. [file DataSheet4.zip › Additional file4/Table S3.docx]

TableS1: Citation cluster naming results from the literature co-citation analysis.

| **Label** | **Ollama** | **LLR** | **LSI** |
| --- | --- | --- | --- |
| 0 | Tumor Microenvironment Analysis | Cancer Cell | Tumor Microenvironment |
| 1 | Single Cell Analysis | Functional Outcome | Single-Cell Analysis |
| 2 | Single Cell Analysis | Mass Cytometry | Mass Cytometry |
| 3 | Tumor Immune Signatures | Arg1 Expression | Tumor-Associated Macrophage |
| 4 | Cancer Immune Dynamics | Metastatic Tumor Ecosystem | Single-Cell RNA-Seq |
| 7 | Tumor Microenvironment Analysis | Bulk Tumor Gene Expression Data | Lung Adenocarcinoma |
| 8 | Immunotherapy Response Prediction | Mesenchymal Stem Cell | Mesenchymal Stem Cell |
| 9 | Tumor Microenvironment Analysis | Precision Medicine | Integrating Bulk |
| 10 | Tumor Microenvironment Dynamics | High-Throughput Single-Cell Microtechnologies | Advances in High-Throughput Single-Cell Microtechnologies |
| 11 | T Cell Dysfunction | Single-Cell RNA-seq | Single-Cell Sequencing |
| 13 | Immune Cell Behavior | Transcriptional Analysis | Single Cell Transcriptional Analysis Reveals Novel Innate Immune Cell Types |
| 14 | Single Cell Analysis | Emerging Single-Cell Technologies | Functional Proteomics |
| 17 | Cellular Signaling Issues | Systems Biology Analysis | Systems Biology Analysis of Heterocellular Signaling |
| 18 | Tcr Identification Challenges | T-Cell Receptor Chain | *Rapid Cloning, Expression, And* Functional Characterization of Paired αβ and γδ T-Cell Receptor Chains from Single-Cell Analysis |
| 19 | Cancer Immune Cell Dynamics | Combating Subclonal Evolution | Resistant Cancer Phenotypep |
