# Supplementary material for: Exploring research trends in cancer immunotherapy via single-cell technologies: a scientometric perspective
Source: Front Immunol. 2025 Aug 22;16:1640224. doi: 10.3389/fimmu.2025.1640224 (PMC12411161; doi:10.3389/fimmu.2025.1640224)
Supplement: Supplementary Table 4 — Keyword co-citation cluster naming results. [file DataSheet5.zip › Additional file5/Table S4.docx]

TableS2: Keyword co-citation cluster naming results.

| Label | Llama | LLR | LSI |
| --- | --- | --- | --- |
| 0 | Cancer Microenvironment Dynamics | Colorectal Cancer | Tumor Microenvironment |
| 1 | Tumor Immune Response | Single-Cell Analysis | Single-Cell RNA |
| 2 | Immune Tumor Microenvironment | Tumor Microenvironment | Single-Cell Analysis |
| 3 | Disease Diagnosis Tools | Longitudinal Multiparameter | Tertiary Lymphoid Structures |
| 4 | Immune Cell Dynamics | Activation | Expression |
| 5 | Tumor Microenvironment Analysis | Dendritic Cells | Hepatocellular Carcinoma |
| 6 | Immunotherapy Response Prediction | Ipilimumab | Machine Learning |
| 7 | Tumor Microenvironment Analysis | Receptor | Receptor |
| 8 | Cancer Immunotherapy | Immune Microenvironment | Immune Microenvironment |
